# The balbyter ant *Camponotus fulvopilosus* combines several navigational strategies to support homing when foraging in the close vicinity of its nest

**DOI:** 10.3389/fnint.2022.914246

**Published:** 2022-09-16

**Authors:** Ayse Yilmaz, Yakir Gagnon, Marcus J. Byrne, James J. Foster, Emily Baird, Marie Dacke

**Affiliations:** ^1^Lund Vision Group, Department of Biology, Lund University, Lund, Sweden; ^2^School of Animal, Plant and Environmental Sciences, University of the Witwatersrand, Johannesburg, South Africa; ^3^Biocenter, University of Wuerzburg, Wuerzburg, Germany; ^4^Neurobiology, University of Konstanz, Konstanz, Germany; ^5^Department of Zoology, Stockholm University, Stockholm, Sweden

**Keywords:** ants, *Camponotus fulvopilosus*, path integration, terrestrial landmarks, short-range navigation, cue weighting

## Abstract

Many insects rely on path integration to define direct routes back to their nests. When shuttling hundreds of meters back and forth between a profitable foraging site and a nest, navigational errors accumulate unavoidably in this compass- and odometer-based system. In familiar terrain, terrestrial landmarks can be used to compensate for these errors and safely guide the insect back to its nest with pin-point precision. In this study, we investigated the homing strategies employed by *Camponotus fulvopilosus* ants when repeatedly foraging no more than 1.25 m away from their nest. Our results reveal that the return journeys of the ants, even when setting out from a feeder from which the ants could easily get home using landmark information alone, are initially guided by path integration. After a short run in the direction given by the home vector, the ants then switched strategies and started to steer according to the landmarks surrounding their nest. We conclude that even when foraging in the close vicinity of its nest, an ant still benefits from its path-integrated vector to direct the start of its return journey.

## Introduction

Path integration is a widespread strategy, employed by many arthropods - including ants, fiddler crabs, beetles, and spiders - to successfully navigate between their home and a feeding place (ants; Müller and Wehner, 1988; fiddler crabs; Zeil, 1998; homing dung beetles Dacke et al., 2020; Yilmaz et al., 2021; spiders; Mittelstaedt, 1983). By keeping track of the directions and distances traveled, and continually integrating these into a single vector, these animals can safely return home even after exploring unfamiliar terrain. Many ants use path integration as their primary system for long-range navigation but can also combine it with learned visual information for improved precision and overall navigational success (Collett et al., 1998; Wehner et al., 2016; Buehlmann et al., 2020a). If these two systems are set into conflict experimentally by, for example, artificially rotating the visual surroundings, most species take a compromise direction when aiming to return to their nests (reviewed in Wehner et al., 2016), resulting in a vector that lies between the two stimuli (Collett, 2012; Wystrach et al., 2015).

The relative weightings of directional guidance from path integration and visual terrestrial landmarks during navigation seems to vary across species, individual experience and specific habitat characteristics (Buehlmann et al., 2020a). For instance, path integration is especially important when naïve ants explore unfamiliar habitats, enabling the inexperienced forager to gradually learn terrestrial landmarks along the new route (Wehner et al., 2004; Müller and Wehner, 2010; Fleischmann et al., 2016). Experienced foragers can then make use of panoramic cues and visual sequences of landmarks along their homeward routes to guide themselves toward the vicinity of their nest, and to finally pin-point the exact location of its entrance (Wehner and Räber, 1979). Ants living in cluttered, landmark-rich environments readily combine directional information from path integration with visual landmarks as soon as they recognize a familiar visual landscape when seen from a new location (Narendra, 2007a), while ants from visually poor habitats rely exclusively on path integration for homing when transferred to an unfamiliar place (Buehlmann et al., 2011).

In contrast to most ants and other homing arthropods, the dung beetle *Scarabaeus galenus* relies primarily (if not exclusively) on path integration to locate its burrow (Dacke et al., 2020; Yilmaz et al., 2021). It is important to note that these short-range navigators (often < 2 m) (Dacke et al., 2020) forage in the landmark-rich African savanna. Whether the apparent inability of the beetle to extract directional information from terrestrial landmarks is related to an incapacity to learn and memorize landmarks, or the possession of a very precise path integrating system, or the short foraging distances involved is currently not known.

In this study, we aimed to define the relative use of landmarks and path integration for short distance navigation in the tawny balbyter ant, *Camponotus fulvopilosus*. This golden-haired species of ants builds its nests under fallen trees, rock slabs or at the base of small bushes in the landmark rich savanna habitats in northern South Africa (Robertson and Zachariades, 1997; see Supplementary Figure 1 for photographic representation of its habitat) and forage individually on the ground for insects or in trees for honeydew (Marsh, 1986; Robertson and Zachariades, 1997, personal observation). In this study, foraging *C. fulvopilosus ants* were trained to feeders placed only 1.25 m away from their nests and then passively displaced around or away from their nest as they attempted to return home. We conclude that even when foraging in the close vicinity of their nest, *C. fulvopilosus* ants benefit from their path-integrated vectors to accurately set out in a homeward direction.

## Materials and methods

### Animals

**FIGURE 1:**
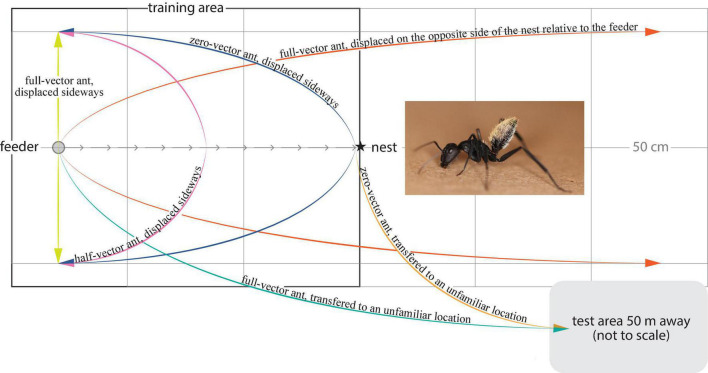
The balbyter ant *Camponotus fulvopilosus* and experimental treatments. The ants were trained to forage on a sucrose solution at a feeder (gray circle), 125 cm away from the nest (star) (Gray arrows, direct path home). Once trained, the ants located the feeder and started to drink from it, were either caught there (full-vector ants, green and yellow), or allowed to fill their gaster and return toward their nest. These ants were then either caught 0.62 m along their path (half-vector ants, pink) or just before they entered their nest (zero-vector ants, blue or orange). Captured ants were then transferred to an unfamiliar test area 50 m away (green), or displaced to a position 50 cm to either side of the feeder (yellow) or to the corresponding position 125 cm on the opposite side of the nest relative to the feeder (orange).

Experiments were performed with *C. fulvopilosus* (Figure 1) in February 2019 on the game farm Stonehenge, near Vryburg, South Africa (26°23′56″S, 24°19′36″E), using three nests located approx. 15 m apart from each other. *C. fulvopilosus* are easily distinguished from other members of the genus by thick yellow hairs on their gaster (Robertson and Zachariades, 1997; Figure 1). Our experiments were limited to minor and medium-sized workers as these are the active foragers within the genus (Josens et al., 2009; Yilmaz et al., 2014, 2016, 2017).

### Activity rhythm measurements

Before the experiments, we monitored the daily activity rhythm of two *C. fulvopilosus* nests for 6 days. A GoPro camera (Hero 6, GoPro, Inc., United States) was placed above each nest and set to capture an image every 10 min from 05.00 to 07.00 and from 19.00 to 20.00, and every 30 min for the remainder of the day. The captured images allowed us to manually identify the number of *C. fulvopilosus* ants present within a 30 cm radius of the nest with increased temporal resolution around sunrise and sunset. Temperature was measured every 30 min (Termometerfabriken Viking AB, Art No: 02038).

### Foraging distance measurements

For an estimate of natural foraging distances for *C. fulvopilosus*, 14 individuals encountered at random were located away from their nest while they were searching for food, provided with sucrose solution and followed back until they reached their nest. The direct distance between their nest and the point where they had been located while foraging measured.

### Behavioral experiments

#### General experimental procedure

A petri dish (3 cm diameter) feeder, filled with meal worms and sucrose solution, was placed on top of a sand covered wooden plate (5 cm × 5 cm) 125 cm away from the nest entrance (Figure 1). Training started at 06:30 and continued until the last ant entered the nest (ca. 19:00). Ants reaching the feeder for the first time were marked with a unique multi-color code (Motip Lackstift Acryl, MOTIP DUPLI GmbH, Haßmersheim, Germany) on the thorax and/or gaster for identification. This allowed us to record the foraging behavior of each individual. Ants that had foraged at least 6 times over a maximum of 2 days, and that ran directly to the feeder and back to the nest without any hesitation, were assigned to one of the 5 experimental conditions. The homebound paths of the ants were recorded from above using a camera (Sony HDR-HC5E Handycam fitted with a 0.66× wide angle lens, Rynox, Japan) mounted on a tripod. The camera set up was present throughout the study. Depending on the experimental condition, the homebound path taken by the ant was either filmed for 4 min or until the ant reached its covered nest entrance.

#### Transfer to an unfamiliar test area

One group of ants was caught individually at the feeder (full-vector ants, Figure 1) by placing an opaque tube over the feeder. The ant and the feeder were then directly transferred to a test area 50 m away, surrounded by an array of different natural landmarks. Here, the feeder was carefully placed on the ground and the opaque tube was removed to release the ant. This whole procedure took approximately 1 min. A second group of ants was instead caught in the opaque tube just before they entered their nest (zero-vector ants, Figure 1) and released in the same test area. As these ants had almost reached their nest when they were caught, their path integrator had nearly been reset to zero.

#### Displacement in the vicinity of the nest

A third group of ants was again caught at the feeder as above (full-vector ants) and displaced 50 cm to the right or to the left from this position (Figure 1). A fourth experimental group of full-vector ants was instead moved 125 cm to the opposite side of the nest relative to the feeder and released 50 cm to the right or to the left of this position (Figure 1). Upon their release, ants from these third and fourth groups set out from a position in space where the nest directions, as dictated by the terrestrial landmarks and those dictated by the path integration, were set in conflict indicating different nest directions. Yet another two groups of ants, the fifth and the sixth group, were caught at 62 cm along their path (half-vector ants) and just before they entered their nest (zero-vector ants) and subsequently released 50 cm to the right or to the left of the feeder (Figure 1). Before each ant was released, the nest was covered with a sandblasted petri dish and the training area was carefully brushed to remove olfactory cues.

### Data analysis

The filmed trajectories of ants were tracked using a custom-made software integrated in Matlab (Mathworks Inc.). The tracks were visualized and analyzed in Julia (Bezanson et al., 2017). Pixel coordinates representing the ant’s position were converted to real world coordinates with the Camera Calibration tool in Matlab. This also accounted for possible distortions in the camera optics. A parametric spline with a factor of 500 and an order of two were used to smooth the coordinates of the ants’ trajectories (Schoenberg, 1971). The vector length for each ant was calculated as the radial (shortest) distance from the release point to the turning point i.e., where the ants changed their direction by a minimum of 60° and then walked along a curved path (see also Dacke et al., 2020). To identify this point in space, the segment of the path containing the turning point was first defined from the derivative of the spline at the spline’s knots (where the sequential smoothing polynomials connected). The exact location of the turning point was then defined as the first point along the identified segment at which the ant’s direction (calculated from the derivative) deviated from the direction at the first of two consecutive knots by more than 60°. The center of search was determined as the mean coordinate of the track, i.e., from the defined turning point until the end of the track or until 4 min had passed from the turning point. The full width at half maximum (FWHM) in Figures 3, 5 refers to the region with the highest search density, calculated as the width of a fitted 2D Gaussian distribution at half of its maximum amplitude.

Vector lengths and the angular distributions of turning points in relation to the ants’ homeward direction were analyzed using Sigmaplot (Systat Software, Inc., San Jose, CA, United States) and Oriana 4.0 (Kovach Computing Services, Anglesey, United Kingdom), respectively. A Rayleigh test was used to test for uniformity of the circular distribution of turning points and a *V*-test was performed to test whether angular positions of the turning points – if significantly different from random – were clustered around the (fictive) nest direction.

## Results

### General description of foraging behavior

The mean natural foraging distance of *C*. *fulvopilosus* workers in the woodland-savanna area examined was 20 m ± 6.3 m (mean ± sd, *n* = 14), with minimum and maximum distances of 8 and 31.3 m, respectively. Even though recruitment behavior with up to 5 individuals was occasionally observed, workers mainly foraged alone. From observation, the inbound and outbound paths of the foraging ants differed between individuals, indicating that the foragers do not follow trail pheromones. This also held true for the first foraging trips of the trained ants.

**FIGURE 2:**
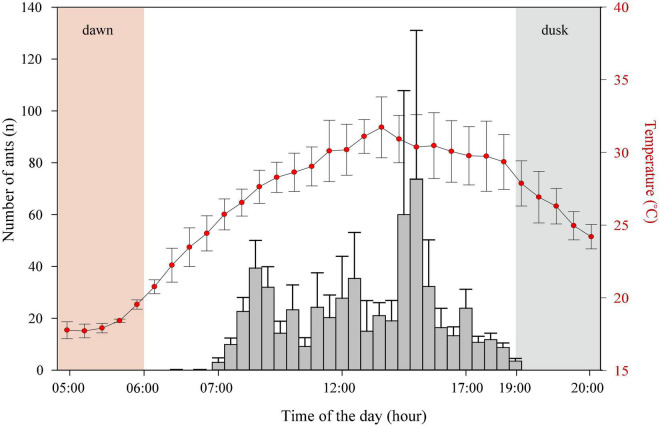
Activity rhythm. The activity period of *Camponotus fulvopilosus* foragers (bars) and air temperature measurements (red dots). Error bars represent standard deviation.

*Camponotus fulvopilosus* started their activity early in the morning between 06.00 and 06.30 with an increase in numbers foraging between 14:30 and 15:00, followed by a gradual decrease from 17:00 to 19:00, after which foragers could no longer be observed (Figure 2).

**FIGURE 3:**
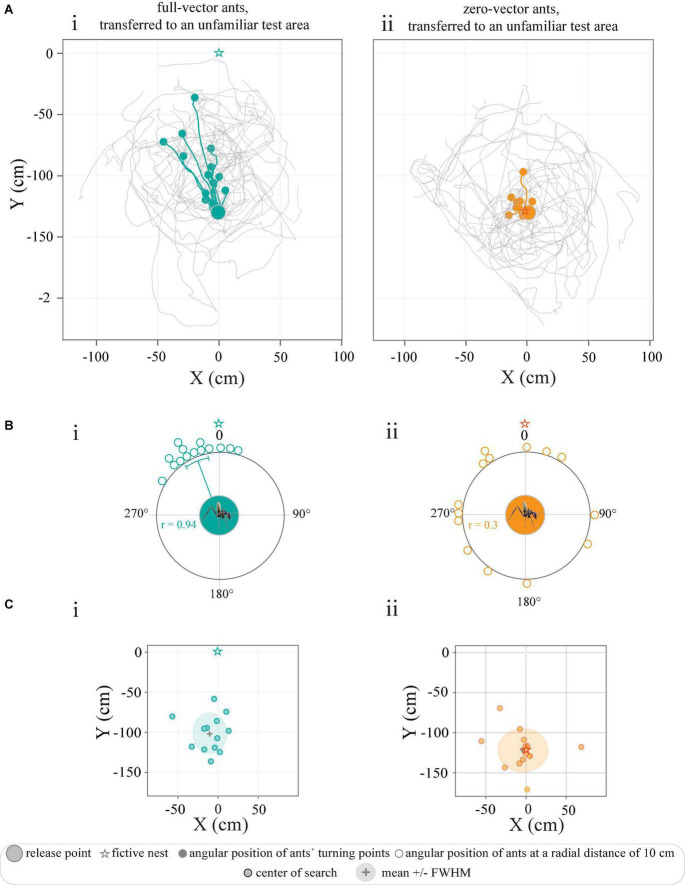
Tracks of full-vector and zero-vector ants transferred to an unfamiliar test area. **(A)** Trajectories represent the paths of ants caught **(i)** at the feeder (full-vector ants, green) or **(ii)** just before they entered their nest (zero-vector ants, orange). Colored trajectories illustrate the paths of individual ants until their turning point (filled circles). Gray paths illustrate their search after the turning point. **(Bi–iii)** Circular graphs represent the angular positions of ants at a radial distance of 10 cm away from the release point in relation to the normalized fictive nest direction (0°). And colored line within the circular graphs indicate mean angles, and the associated sectors represent the 95% confidence interval of the mean. r represents mean vector length. **(C)** The center of the search paths described with respect to the location of the fictive nest (star). Gray crosses and shaded colored areas mark the mean and the full width at half maximum (FWHM) of the data.

### Ants transferred to an unfamiliar test area navigate by path integration

When caught at the feeder and transported to an unfamiliar test area 50 m away (Figure 1), the full-vector ants were still drinking, and showed no sign of disturbance by being covered and moved. When full, as indicated by a swollen gaster, the ants quickly ran in the direction of their fictive nest (i.e., the position of their nest had it been moved with them) for 41.5 ± 25.8 cm (Figure 3Ai) before they made a turn (defined as their turning points) and initiated a systematic search. An analysis of the positions of the ants at a radial distance of 10 cm from the release point clearly revealed that the initial directions traveled were clustered in the direction of the fictive nest (*P* < 0.0001, *r* = 0.94, Rayleigh test, *P* < 0.0001, *V*-test, μ: 335.9° ± 6.0°, *V* = 0.85, *n* = 14) (Figure 3Bi). This also held true for the spatial distribution of their turning points (*P* < 0.0001, *r* = 0.94, Rayleigh test, *P* < 0.0001, *V*-test, μ: 344.9° ± 5.7°, *V* = 0.91, *n* = 14). The subsequent searches centered 25 cm away from the release point (*x*-axis: −9.9 ± 18.7 cm, *y*-axis: −100.9 ± 22.1 cm, Figure 3Ci).

In contrast, the zero-vector ants released in the same test area were no longer oriented to a specific (fictive nest) direction (Figure 3Aii). The center of search in this group was rather located around their point of release (0, −125 cm), that is, around the fictive nest position as indicated by their nearly zeroed path-integrator (*x*-axis: −3.5 ± 26.9 cm, *y*-axis: −121.7 ± 23.2 cm) (Figure 3Cii). Analysis of the angular distribution of these zero-vector ants when at a radius of 10 cm away from their point of release further confirmed that this group of ants were indeed randomly oriented (*P* = 0.1, *r* = 0.3, Rayleigh test, *n* = 14) (Figure 3Bii). Overall, the results indicate that *C. fulvopilosus* workers relied on path integration for homing when released in an unfamiliar area. The non-directed behavior of the zero-vector ants also confirms that landmarks (distant or local) did not provide any guidance from this presumed unfamiliar point of release.

### Path integration guides initial homing even when foraging only 125 cm away from the nest

**FIGURE 4:**
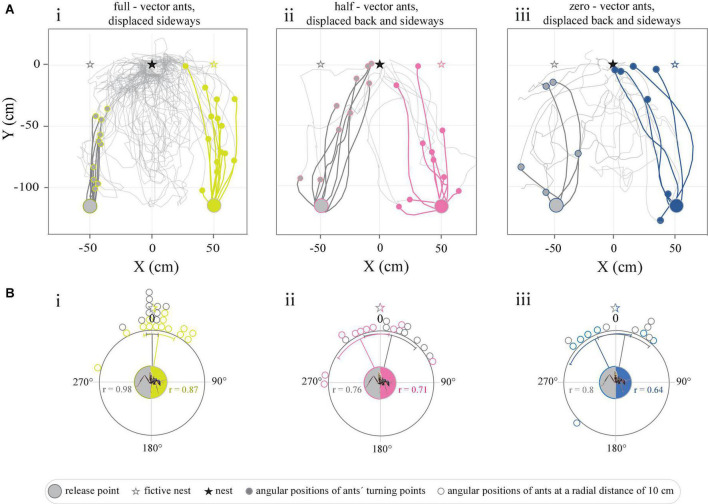
Tracks of full-vector, half-vector, and zero-vector ants displaced within the vicinity of the nest. **(A)** Trajectories represent the paths traveled upon the release of ants captured **(i)** at the feeder (full-vector ants, dark gray-yellow), **(ii)** midway on their route home (half-vector ants, dark gray-pink), or **(iii)** just before they entered their nest (zero-vector ants, dark gray-blue). Light gray paths illustrate their search. **(Bi–iii)** Circular graphs represent the angular positions of ants at a radial distance of 10 cm from the release point in relation to the normalized fictive nest direction (0°). Gray and colored lines within the circular graphs indicate mean angles, associated sectors represent the 95% confidence interval of the mean. r represents mean vector length.

Ants caught at the feeder and displaced 50 cm sideways to the right or to the left of it, first set off in the direction of their fictive nest (straight ahead) and ran in this direction for 48.05 ± 20.8_*left*_ and 64.7 ± 32.1_*right*_ cm before adjusting their bearings toward the actual position of the real nest (Figure 4Ai and Supplementary Figure 2). As expected, an analysis of the positions of the ants at a radial distance of 10 cm from the release point revealed that the initial directions traveled by the ants were clustered in the direction of the fictive (rather than the real) nest (*P*_*left*_ < 0.0001, *r* = 0.98, Rayleigh test, *P*_*left*_ < 0.0001, *V*-test, μ: 359.6° ± 3.9°, *V* = 0.98, *n* = 10; *P*_*right*_ < 0.0001, *r* = 0.87, Rayleigh test, *p*_*right*_ < 0.0001, *V*-test, μ: 7.2 ± 8.7°, *V* = 0.87, *n* = 14) (Figure 4Bi). A similar pattern was recorded for the relative spatial distribution of the ants’ turning points (*P*_*left*_ < 0.0001, *r* = 0.99, Rayleigh test, *P*_*left*_ < 0.0001, *V*-test, μ: 5.9 ± 2.47°, *V* = 0.98, *n* = 10; *P*_*right*_ < 0.0001, *r* = 0.97, Rayleigh test, *p*_*right*_ < 0.0001, *V*-test, μ: 359. ± 4.47°, *V* = 0.96, *n* = 14). From the turning point onward, the paths of the ants curved toward their real nest (Figure 4Ai and Supplementary Figure 2). This suggests that the homing ants first set out in the direction indicated by their path integrator, but then changed their bearings to agree with the directional information provided by its well-known surroundings.

**FIGURE 5:**
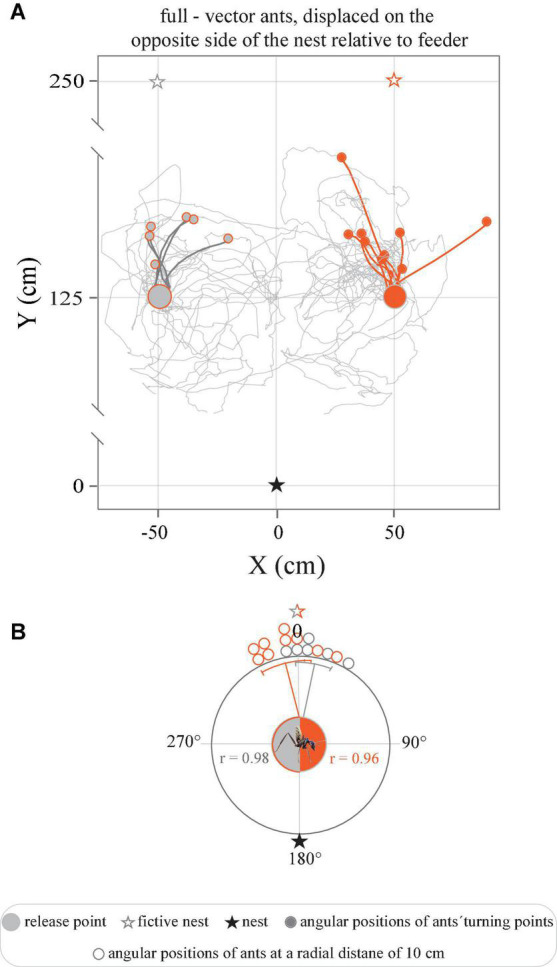
Tracks of full-vector ants displaced from the feeder to the opposite side of the nest. **(A)** Trajectories represent the paths traveled upon the release of ants captured at the feeder and moved to the opposite side of the nest, with a 50 cm displacement right or left relative to the feeder. **(B)** Circular graphs represent the angular positions of ants at a radial distance of 10 cm from the release point in relation to the normalized fictive nest direction (0°). Gray and orange lines indicate mean angles, associated sectors represent the 95% confidence interval of the mean. r represents mean vector length.

When instead moved from the feeder to a corresponding position, displaced 50 cm to the right or to the left of the feeder, on the opposite side of the nest (Figure 1) the ants showed an even greater conflict between the directional information provided by their path-integrator and local landmarks. The full-vector ants nonetheless initially set off in the direction of the fictive nest, i.e., away from their real nest, and ran in this direction for 23.9_*left*_ ± 9.9 cm and 30.9_*right*_ ± 14.1 cm (Figure 5A). Consequently, an analysis of the positions of the ants at a radial distance of 10 cm from the release point clearly revealed that the initial directions traveled by the ants were clustered in the direction of the fictive nest, i.e., directly away from the real nest (Figure 5B) (*P*_*left*_ < 0.0001, *r* = 0.98, Rayleigh test, *P*_*left*_ < 0.0001, *V*-test, μ: 7.4 ± 6.2°, *V* = 0.97, *n* = 6); (*P*_*right*_ < 0.0001, *r* = 0.96, Rayleigh test, *P*_*right*_ < 0.0001, μ: 351.0 ± 6.3°, *V* = 0.95, *n* = 9). This also held true for the relative spatial distribution of their turning points (*P*_*left*_ < 0.0001, *r* = 0.90, Rayleigh test, *P*_*left*_ < 0.0001, *V*-test, μ: 11.2 ± 14.0°, *V* = 0.88, *n* = 6); (*P*_*right*_ < 0.0001, *r* = 0.88, Rayleigh test, *P*_*right*_ < 0.0001, μ: 350.3 ± 11.5°, *V* = 0.86, *n* = 9). The ants then adjusted their bearings toward the true position of their real nest.

Unlike the full-vector ants, the trajectories of half-vector ants (caught after running half of their home vector then displaced 50 cm sideways to the left or right of the feeder) were instead biased toward the position of the real nest (Figure 4Aii). An analysis of the angular distribution of the half-vector ants at a radius of 10 cm away from their point of release further confirmed that this group of ants were indeed oriented toward the real nest (*P*_*left*_ = 0.003, *r* = 0.76, Rayleigh test, *P*_*left*_ < 0.01, *V*-test, μ: 334.3° ± 16.6°, *V* = 0.74, *n* = 9; *P*_*rightt*_ = 0.004, *r* = 0.71, Rayleigh test, *P*_*right*_ < 0.001, *V*-test, μ: 13.3° ± 17.4°, *V* = 0.64, *n* = 10) (Figure 4Bii). As for half-vector ants, the trajectories resulting from displaced zero-vector ants (caught just before entering their nest and again displaced 50 cm sideways to the left and right of the feeder), were biased toward the real nest direction (*P*_*left*_ = 0.05, *r* = 0.65, Rayleigh test, *P*_*left*_ = 0.01, *V*-test, μ: 13.6° ± 20.3°, *V* = 0.57, *n* = 6; *P*_*right*_ = 0.01, *r* = 0.8, Rayleigh test, *P*_*right*_ < 0.01, *V*-test, μ: 334.3° ± 21.9°, *V* = 0.78, *n* = 7) (Figures 4Aiii,Biii).

## Discussion

After having repeatedly foraged only 125 cm away from its nest, *C. fulvopilosus* ants displaced at a feeder first set out in the direction given by their path integrator (Figure 4). Then, approximately 50 cm down the path, they instead adjusted their course according to surrounding landmarks and reliably pin-pointed the position of their nest entrance. These results clearly demonstrate that both navigational systems are actively engaged in the balbyter ants even when foraging only slightly more than 1 m away from their nest.

### *Camponotus fulvopilosus* relies on path integration to initiate its return journey when on an unfamiliar territory

After transferring *C. fulvopilosus* foragers, with an expected home-vector of approximately 1.25 m, to a distant test area with a new array of landmarks, the ants ran 41.5 cm on average in the direction of their fictive nest before they started to run along a curved track, resembling the well documented search patterns of other ant species (see for example Wehner and Srinivasan, 1981; Schultheiss and Cheng, 2011; Figure 3Ai). This clearly demonstrated that *C. fulvopilosus* ants develop and rely on their home-vectors for homing even after repeatedly foraging over the relatively short distance of 125 cm.

The finding that the full-vector *C. fulvopilosus* ants did not run off their entire home vector before they engaged in a systematic search is similar to earlier observations in other ant species inhabiting landmark rich habitats (*Gigantiops destructor*, Beugnon et al., 2005; *Formica japonica*, Fukushi, 2001; *Melophorus bagoti*, Narendra, 2007a,b; Buehlmann et al., 2011; Cheng et al., 2012; Schultheiss et al., 2016), but contrasts to the strong adherence to the path-integrated vector in ants living in landmark-poor environments (*Cataglyphis* fortis, Buehlmann et al., 2011; see also *Melophorus sp*. in Schultheiss et al., 2016). The early break-off from the home-vector in *C. fulvopilosus* ants thus further supports the hypothesis that the adherence to the home vector is inversely correlated with the density of vegetation in the habitat of the species (Buehlmann et al., 2020a) and likely reflects a functional adaptation of the navigational system to maximize the probability of finding the nest (Narendra, 2007b). Interestingly, the homing dung beetle *Scarabaeus galenus*, active in the same landmark-rich habitat as *C. fulvopilosus* ants, runs off as much as 95% of their 1.25 m home-vector, before initiating a winding search for their burrows under identical experimental conditions (Dacke et al., 2020). This strong adherence to the path-integrated vector is likely explained by the apparent inability of the beetles to navigate by the use of landmarks (Dacke et al., 2020).

The transfer of zero-vector ants to the same distant test-area gained an entirely different result; these ants ran in random directions from their point of release and initiated their systematic searches around the same point in space, i.e., close to the fictive position of their nest (Figures 3Aii,Cii). Given the unfamiliarity of these ants with the surrounding visual scene at the test area, this outcome is not surprising but nicely demonstrates that the testing area – chosen to lie outside the foraging range of the workers – did not provide the transferred ants with any navigationally relevant visual information to point them in a nest-ward direction.

### *Camponotus fulvopilosus* relies on path integration to initiate its return journey when foraging in the close vicinity of the nest

When displaced 50 cm sideways in the familiar surroundings around their nest, full-vector *C. fulvopilosus* foragers still followed their home vectors (i.e., they ran directly toward their fictive nests) for the first 64 ± 32 cm (right displacement) and 38 ± 16 cm (left displacement) of their return journeys (Figure 4Ai). After this point, they headed toward the real nest (Figure 4Ai), presumably guided by the landmarks around them. That is, the full-vector ants initially set out in the direction given by their home-vectors, and then switched to following the terrestrial landmark information further down the homeward route. When released at the same locations (50 cm left or right of the feeder, see Figure 1), displaced zero-vector ants, that had no vector or a very short vector to help point them in the correct direction, at first were more spread in their initial bearings, but were still all able to return home (Figure 4Aiii), most likely by using landmarks. This suggests that full-vector *C. fulvopilosus* ants, when displaced to the exact same two locations (50 cm sideway from the feeder) could also have returned home by relying solely on landmarks, but instead used their path-integrated vector to guide the initial segments of their return paths. This also held true for full-vector ants displaced from the feeder to the opposite side of the nest, that were observed to initially run directly away from their nest (Figures 1, 5).

### The weight given to path-integration and landmarks shifts with the relative length of the home vector

When captured midway on their route home (half-vector ants) and displaced to either of two locations (50 cm sideways from the feeder, see Figure 1) ants were – unlike the full-vector ants – already initially biased toward their real nest (Figures 4Aii,Bii). This suggests that after running off half of their home vector, the relative weight given to the directional information provided by local landmarks had increased. This corresponds with previous findings with *Cataglyphis fortis* ants that had run off 60% of their home vector and consequently responded more strongly to learnt visual landmarks than those ants that had run off only 10% of their home vector (Buehlmann et al., 2018). It has further been shown in *Melophorus bagoti* and *C. nodus* ants that the weighting given to landmarks increases with accumulated visual experience (Freas and Cheng, 2017; Fleischmann et al., 2018). In general, it is believed that for most ants the relative weight given to directional information is biased toward the more reliable source of information (Reid et al., 2011; Collett, 2012; Legge et al., 2014; Wystrach et al., 2015; Wehner et al., 2016). This suggests that at the start of the short return journey (1.25 m), *C. fulvopilosus* ants weight directional information provided by the home vector more strongly than information provided by landmarks. Whether this is also the case in other ant species when foraging in the close vicinity of their nests remains to be shown.

### Several navigational strategies support homing when foraging in close vicinity to the nest

Our study suggests that *C. fulvopilosus*, like many other ant species, optimizes its homing strategy by weighting the relationship between directional information provided by path integration and visual landmarks, even when foraging over the relatively short foraging distances evaluated in this study. The weighting relationship of these two navigational strategies is shaped by species specific-visual habitat characteristics (see also Cheng et al., 2012), with ants living in landmark-rich habitats relying less strongly on path integration when transferred to an unfamiliar test area or when familiar landmarks were removed (Fukushi, 2001; Beugnon et al., 2005; Narendra, 2007b; Schultheiss et al., 2016). The inclusion of landmark information into a navigational toolkit increases the precision of the navigational system as a whole (Buehlmann et al., 2020a) but comes at the cost of energy and requires an improved cognitive capacity with more complex neuronal implementation (Rössler, 2019; Buehlmann et al., 2020b; Kamhi et al., 2020). Many ant species perform learning walks, including frequent rotations around their own body axis, for several days to establish a *de novo* long-term memory of stable nest related visual cues (Fleischmann et al., 2016, 2017; Jayatilaka et al., 2018; Zeil and Fleishmann, 2019). Whether *C. fulvopilosus* ants also perform learning walks or acquire their landmark information by some other way is yet unknown, but the ants convincingly demonstrate the cognitive capacity to learn and process this type of navigation-relevant visual information. Starting their short return journeys in the direction dictated by their home-vector probably places them in the best possible position to utilize the stored memory of landmarks in the most efficient way.

Interestingly, homing beetles – which inhabit the same environment as the *C. fulvopilosus* ants – show no indication of rotations (to look back at their burrow), learning walks of increasing distance or any other signs of the inclusion of landmarks for navigational purposes (Dacke et al., 2020). In addition, the beetles engage in an extended, circling search to locate their semi-permanent burrows in response to 50 cm sideway displacements at the feeder (Dacke et al., 2020). Equipped with the ability to correct for these types of passive displacements by using landmarks, an ant will locate its nest much more efficiently (see Figure 4 in this study and Figure 5 in Dacke et al., 2020). These differences in navigation strategies likely reflect the ecological needs of these two groups of navigators; an ant without a nest and a colony perishes rapidly, while a solitary beetle can dig a new burrow within the next hour with little fitness cost.

In summary, the combination of path-integration and landmarks for navigation effectively guides *C. fulvopilosus* – and other ants – to quickly orient themselves in a nest-ward direction. Further down the path, known landmark memories allow them to locate their nests more accurately even if displaced from their intended route (experimentally or by a strong gust of wind). Our results thus emphasize the benefit of several navigational strategies to support homing even when foraging only a couple of meters away from home.

## Data availability statement

The raw data supporting the conclusions of this article will be made available by the authors, without undue reservation.

## Author contributions

AY, EB, and MD designed the experiments. AY, EB, MD, MB, and JF performed the experiments. YG designed the software for data analysis. AY analyzed the data. AY and MD wrote and revised the manuscript. All authors have read, revised, and agreed to the published version of the manuscript.
