# Supplementary material for: The balbyter ant Camponotus fulvopilosus combines several navigational strategies to support homing when foraging in the close vicinity of its nest
Source: Front Integr Neurosci. 2022 Sep 16;16:914246. doi: 10.3389/fnint.2022.914246 (PMC9523141; doi:10.3389/fnint.2022.914246)
Supplement: Supplementary file 1 [file Data_Sheet_1.docx]

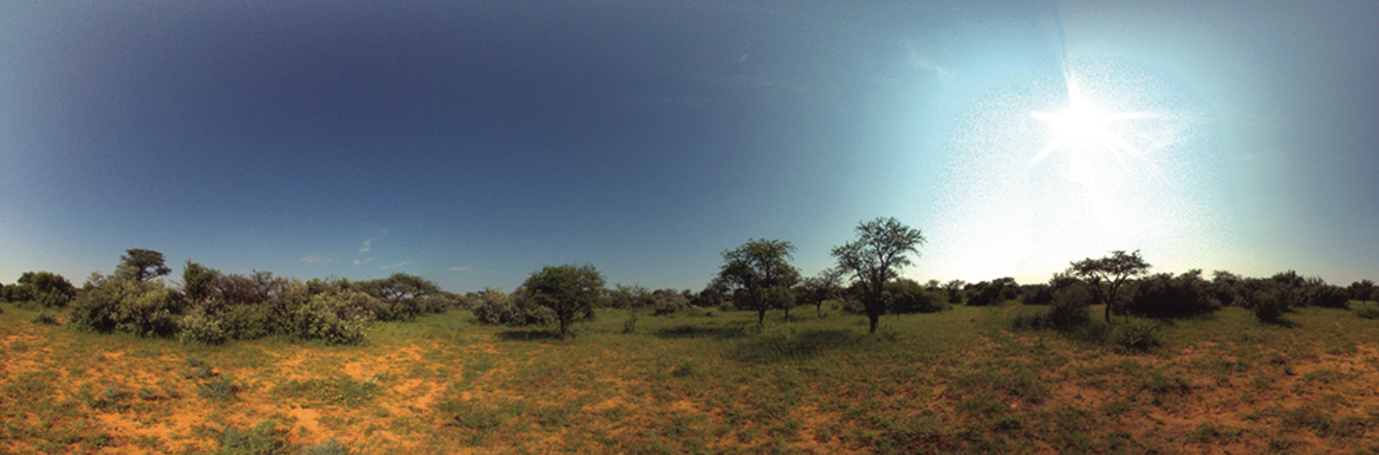


**Figure S1.** The natural woodland-savanna habitat of *Camponotus fulvopilosus.*


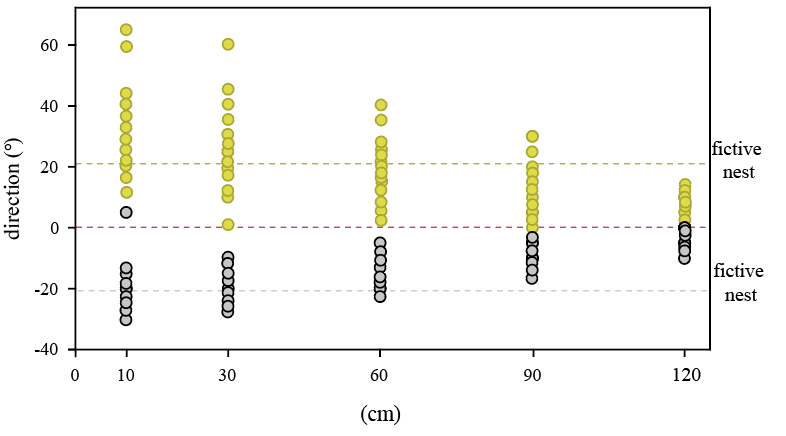


**Figure S2.**  Full-vector *Camponotus fulvopilosis* ants were displaced 50 cm to the right or to the left at their feeder 1.25 m away from the nest. The angular distribution of the displaced ants in relation to the real nest (0°, red line) was recoded at radial distances of 10, 30, 60, 90 and 120 cm from the release point. Data from right displaced ants is shown in yellow, data from left displaced ants is shown in grey. The displaced ants initially followed a bearing as indicated by their path integrator, i.e towards their fictive nest (dashed lines). They then gradually adjusted their bearings towards the real nest (red line).
